# Human Rabies and Rabies in Vampire and Nonvampire Bat Species, Southeastern Peru, 2007

**DOI:** 10.3201/eid1508.081522

**Published:** 2009-08

**Authors:** Gabriela Salmón-Mulanovich, Alicia Vásquez, Christian Albújar, Carolina Guevara, Alberto Laguna-Torres, Milagros Salazar, Hernan Zamalloa, Marcia Cáceres, Jorge Gómez-Benavides, Victor Pacheco, Carlos Contreras, Tadeusz Kochel, Michael Niezgoda, Felix R. Jackson, Andres Velasco-Villa, Charles Rupprecht, Joel M. Montgomery

**Affiliations:** US Naval Medical Research Center Detachment, Lima, Peru (G. Salmόn-Mulanovich, C. Albújar, C. Guevara, V.A. Laguna-Torres, T. Kochel. J.M. Montgomery); Johns Hopkins Bloomberg School of Public Health, Baltimore, Maryland, USA (G. Salmόn-Mulanovich); Universidad Nacional Mayor de San Marcos, Lima (A. Vásquez, V. Pacheco); University of Texas Medical Branch, Galveston, Texas, USA (M. Salazar); Instituto Nacional de Salud, Lima (H. Zamalloa); Dirección de Salud Madre de Dios, Puerto Maldonado, Peru (M. Cáceres, C. Contreras); Dirección General de Epidemiologia, Lima (J. Gómez-Benavides); Centers for Disease Control and Prevention, Atlanta, Georgia, USA (M. Niezgoda, F.R. Jackson, A. Velasco-Villa, C. Rupprecht)

**Keywords:** Rabies virus, rabies, viruses, outbreak investigation, reservoir, vampire bats, Peru, dispatch

## Abstract

After a human rabies outbreak in southeastern Peru, we collected bats to estimate the prevalence of rabies in various species. Among 165 bats from 6 genera and 10 species, 10.3% were antibody positive; antibody prevalence was similar in vampire and nonvampire bats. Thus, nonvampire bats may also be a source for human rabies in Peru.

Rabies is one of the better known encephalitis viruses of the family *Rhabdoviridae* and genus *Lyssavirus*. In the sylvatic cycle, this infection is maintained as an enzootic disease in several species, such as foxes, raccoons, and bats. The hematophagous bat, *Desmodus rotundus,* is the main vector and reservoir for sylvatic human rabies in South America (*1*), in contrast with other areas in the world where dogs serve as the main source of infection for humans in the urban cycle.

## The Study

During December 2006–March 2007, a total of 23 human sylvatic rabies cases occurred in Puno (n = 6) and Madre de Dios (n = 17), Peru. The affected population consisted mainly of gold miners and their families who relocate to the area during the rainy season for small-scale mining before returning to their original towns for agricultural activities during the rest of the year. A vampire bat (*D. rotundus*) rabies virus variant was identified from clinical samples of deceased patients (*2*).

After the rainy season ended, a team from the US Naval Medical Research Center Detachment, the Ministerio de Salud (Peruvian Ministry of Health), the Servicio Nacional de Sanidad Agraria (Agricultural Health Service), and the Museo de Historia Natural (Natural History Museum) from the Universidad Nacional Mayor de San Marcos (San Marcos University) traveled to the area to conduct bat collections. The objective of the survey was to identify the prevalence of rabies infection among hematophagous (vampire) and nonhematophagous bats and to assess the distribution of bat genera within the outbreak area.

We sampled 2 study sites (A and B) for this survey. The first collection was conducted in the region of Madre de Dios (location A, 13° 7′53.53′′S, 70°24′28.27′′W) from May 2 through May 4, 2007. The second site was located in the region of Puno (location B, 13°15′29.57′′S, 70°19′39.14′′W) and sampled from May 6 through May 10, 2007 (Figure). Both trapping sites were located within 1 km of reported rabies cases in humans.

**Figure:**
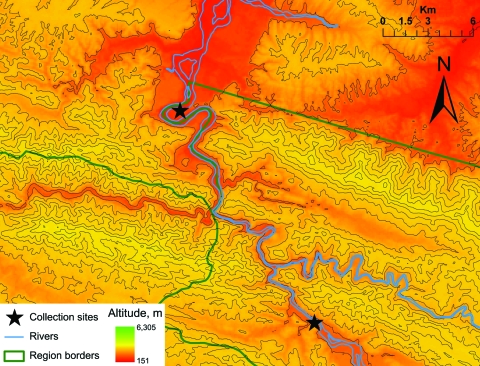
Bat sampling areas, southeastern Peru, 2007.

Bats were collected by using mist nets. These nets were situated along the river banks, in secondary forests, and in manioc and banana plantations. In addition to the above habitats, bats in location B were collected in cattle grazing areas and in 2 caves. Mist nets were set out every afternoon before dusk, checked every 1.5 hours, and closed at midnight, after 7 hours.

Bats were removed from mist nets by using protective leather gloves. Each animal was placed inside a canvas bag, transported to a processing location, and kept until the following morning when they were processed as previously described (*3*). Brain tissues and blood samples collected on necropsy were kept in a liquid nitrogen container for storage and shipping to the Centers for Disease Control and Prevention (CDC) in Atlanta, GA, USA. Carcasses were tagged and stored in formalin for shipment to the Museo de Historia Natural in Lima for positive species identification.

Blood samples processed at CDC were tested for rabies virus–specific antibodies by using a rapid fluorescent focus inhibition test (RFFIT). Brain stems were tested by direct fluorescence antibody (DFA) for evidence of active disease. Antibody prevalence was calculated by using the binomial exact method; antibody prevalence rates between bat species, location, and genera were compared by using the χ^2^ and Fisher exact tests. All analyses were performed with Stata 10.0 (StataCorp, College Station, TX, USA).

A total of 195 bats were captured. All brain tissues were negative for rabies infection by DFA. Sufficient quantity of serum for RFFIT was available from only 165 (85%) of the sampled animals, which were included in this study. The bats that were collected represented 6 genera, including 10 species; 103 (62%) were females; 62 (38%), males; 25 (15%), juveniles; 140 (85%, adults; and 125 (76%) were *Carollia* spp. (Table 1). One hundred thirty-seven animals (83%) were collected from community B. All vampire bats (n = 7) were collected in this location as well as other non-*Carollia* insectivorous and frugivorous bats (p = 0.001).

**Table 1:** Bat species collected for rabies testing, southeastern Peru, 2007

Species	Frequency (%)
*Carollia perspicillata*	95 (57.58)
*Artibeus lituratus*	18 (10.91)
*Carollia brevicauda*	17 (10.30)
*Carollia benkeithi*	13 (7.88)
*Desmodus rotundus*	7 (4.24)
*Anoura caudifera*	6 (3.64)
*Choeroniscus minor*	4 (2.42)
*Artibeus obscurus*	2 (1.21)
*Dermanura anderseni*	2 (1.21)
*Artibeus planirostris*	1 (0.61)
Total	165 (100)

Bats from the genus *Carollia* were collected more frequently from natural, non-disturbed refuges (e.g., creeks, caves), while other insectivorous, frugivorous, and vampire bats were found in more visibly disturbed or modified foraging areas (e.g., plantations, cattle farms) (p<0.001). Seventeen bats were antibody positive to rabies virus (cut-off value 0.5 IU), for an antibody prevalence of 10.3% (95% confidence interval 6.1–16.0). Antibody prevalence was similar (p = 1.000) among vampire bats (1/7, 14%), *Carollia* spp. (12/125, 10%), and other nonvampire bat genera (*Uroderma*, *Sturnira*, *Platyrhinus,* and *Artibeus*) (4/33, 12%) (Table 2). No statistical differences were found between antibody prevalence and sex (p = 0.111), age (p = 0.078), habitat (p = 1.000), or collection site (p = 1.000) using Fisher exact test.

**Table 2:** Rabies antibody prevalence, vampire and nonvampire bats, southeastern Peru, 2007*

Variables	No. positive/ no. sampled	p value
Sex		0.111
F	14/103	
M	3/62	
Age		0.078
Subadult	0/25	
Adult	17/140	
Genera		0.685
*Desmodus* sp.	1/7	
*Carollia* sp.	12/125	
Others	4/33	
Isolation location		1.000
A	3/28	
B	14/137	
Refuges and foraging areas†		1.000
Natural	7/77	
Disturbed	8/79	

*n = 165. Overall prevalence was 10.3 (95% confidence interval 6.1–16.0). †Information on refuges and foraging areas is missing for 9 individuals, including 2 positive *Carollia perspicillata* bats.

## Conclusions

In recent years, cases of rabies among humans in urban areas (transmitted by domestic animals) have declined considerably in the Americas. This is likely the result of an aggressive initiative by the member states of the Pan American Health Organization (PAHO) to eliminate urban rabies in the Americas (*4*). Consequently, rabies infections acquired by humans from wild animals, or sylvatic rabies, now represents the primary source for human infection in the region (*1*), with a similar trend for Peru (*5*).

Haematophagous bats, including *D. rotundus*, are usually the species associated with sylvatic bat rabies outbreaks in South America, but little is known about the role of nonhematophagous bats. An investigation in Chile found that nonvampire bats may in fact serve as adequate vectors of sylvatic rabies and even confirmed a single human infection of nonvampire-bat variant rabies linked to a nonhematophagous bat (*6*,*7*). Likewise, PAHO reported 3 cases of sylvatic rabies transmitted by nonhematophagous bats in 2004 (*4*). This may suggest that insectivorous and frugivorous bats have a more specific role in the transmission of rabies virus in South America.

The antibody prevalence of 10.3% found in our study is concordant with the 37% antibody rates found in nonhematophagous bats in Colima, Mexico (*8*) and 7.6% and 12.8% antibody prevalence among vampire and nonvampire bats in Grenada and Trinidad, respectively (*9*). Although the transmission of rabies virus seems to occur very early in life (*10*,*11*), our study did not demonstrate evidence of antibodies to the virus among juvenile bats. Likewise, the distribution of gender suggested higher antibody prevalence among females, although the finding was not significant, perhaps due to small sample size.

The different distribution of bat species may be related to food availability, which would explain why *D. rotundus* bats were found near cattle farms and the more ubiquitous distribution of *Carollia perspicillata* bats, which feed primarily on fruit in addition to pollen and insects. The bat species collected in our study have been previously found in areas at similar altitudes in Peru; therefore, their distribution in this area follows a regular pattern (*12*,*13*). Additionally, the lack of surveillance for bat populations in the area prevents further inference about a possible source of infection among these bat populations. Although none of the bats tested in our investigation had active rabies infections, both vampire and nonvampire bats had evidence of antibodies to rabies virus, which perhaps suggests ongoing cross-species transmission (spillover) among multiple bat species.

Peru and other South American countries should enforce the comprehensive, more aggressive preventive measures suggested at the XI Reunion de Directores de los Programas Nacionales de Control de Rabia en America Latina (Meeting of the Directors of National Rabies Control Programs in Latin America) for certain prioritized areas. These activities include preexposure prophylaxis, surveillance, closer coordination with local animal health authorities, and community education.
